# Efficacy and safety of radiofrequency catheter ablation in younger children with ventricular arrhythmias

**DOI:** 10.3389/fped.2025.1555830

**Published:** 2025-04-23

**Authors:** Ziyan Dong, Zhen Zhen, Xia Yu, Lang Cui, Wei Shao, Li Lin, Lu Gao, Yue Yuan

**Affiliations:** ^1^ Department of Pediatrics, Beijing Tiantan Hospital, Capital Medical University, Beijing, China; ^2^ Department of Cardiology, Beijing Children’s Hospital, National Center for Children’s Health, Capital Medical University, Beijing, China

**Keywords:** ventricular arrhythmias, radiofrequency catheter ablation, low age, children, safety and efficacy

## Abstract

**Background and Aims:**

To compare the clinical characteristics of ventricular arrhythmias (VAs) in younger vs. older children, and to explore the safety and efficacy of radiofrequency catheter ablation (RFCA) in the treatment of VAs in young children.

**Methods:**

General data, surgical data, and prognosis of all children with VAs who underwent RFCA at our hospital between 1 January 2005 and 31 December 2022 were retrospectively analysed. Patients were divided into two groups according to age: younger (<4 years); and older (≥4 years).

**Results:**

Data from 762 children with VAs were analysed, including 96 younger children (3.47 ± 0.75 years, 16.74 ± 3.98 kg) and 666 older children (9.44 ± 2.86 years, 38.85 ± 15.11 kg). Compared with the older group, there were more premature ventricular beats with ventricular tachycardia, earlier surgery, and more ablations under x-ray fluoroscopy in the younger group. The right ventricular outflow tract was the most common arrhythmia focus in both groups, followed by the tricuspid annulus in the younger group and the left ventricular septum in the older group. The acute success rates for younger and older children were 99.0% and 98.6%, respectively. The recurrence and complication rates in the two groups were similar, and there was no significant difference in radiation dose.

**Conclusions:**

The safety and efficacy of RFCA in the treatment of younger children with VAs were similar to those of older children. RFCA appears to be a viable therapeutic option for young children with drug-resistant VAs.

## 1 Introduction

Ventricular arrhythmias (VAs) are a group of ectopic heart rhythms that originate from the ventricle, including premature ventricular contractions (PVCs) and ventricular tachycardia (VT). The right ventricular outflow tract is the most common origin, followed by the left ventricular septal area; a few originate from the His bundle region, left ventricular outflow tract, ventricular free wall, tricuspid valve, and fascicle. Younger children with VAs often present with sudden pallor, refusal of food or milk, vomiting, and/or lethargy. Those with sustained VAs are more likely to experience tachycardia, cardiomyopathy, and heart failure than older children. Radiofrequency catheter ablation (RFCA) is a radical treatment for VAs and the first-line treatment for idiopathic VAs among school-age children. However, in previous studies and guidelines (1, 2), age <4 or 5 years if age was considered to be a criterion for high-risk RFCA procedures. Few studies have described experiences with RFCA in younger children with VAs and, moreover, most were small, single-centre studies and case reports. In this investigation, we retrospectively analysed data from children with VAs who underwent RFCA at our hospital between 1 January 2005 and 31 December 2022. This study aimed to compare the safety and efficacy of RFCA in younger vs. older children.

## 2 Materials and methods

### 2.1 Study subjects

Data from all children and adolescents <18 years of age diagnosed with VAs who were scheduled to undergo RFCA at Beijing Children's Hospital (Beijing, China) between 1 January 2005 and 31 December 2022, were included. Data from patients who underwent primary and secondary ablation were assessed separately to avoid bias. The children were divided into two groups according to age: younger (<4 years); and older (≥4 years).

Inclusion criteria: VTs or PVCs (>10,000/24 h) with or without clinical symptoms, diagnosed using electrocardiography, echocardiography, physical examination, and recurrent tachyarrhythmias (≥2); drug treatment (e.g., amiodarone, sotalol, metoprolol, lidocaine, verapamil) was ineffective, could not be tolerated long-term, or family members requested surgical treatment; all children <4 years were symptomatic, with specific risk factors (e.g., tachycardia-induced cardiomyopathy, sustained VT, left ventricular dysfunction, history of syncope, heart failure); and all guardians signed informed consent to comply with the 2013 Declaration of Helsinki.

Exclusion criteria: Surgical or anaesthesia-related contraindications; complicated with severe organic heart disease (such as congenital heart disease, ischaemic heart disease); and combined with other systemic diseases such as endocrine or rheumatic immune diseases.

### 2.2 Study protocol

#### 2.2.1 Preoperative preparation

All children discontinued antiarrhythmic drugs for 5 half-lives before electrophysiological examination and all fasted for >8 h before surgery.

#### 2.2.2 Surgical process

**Figure 1:**
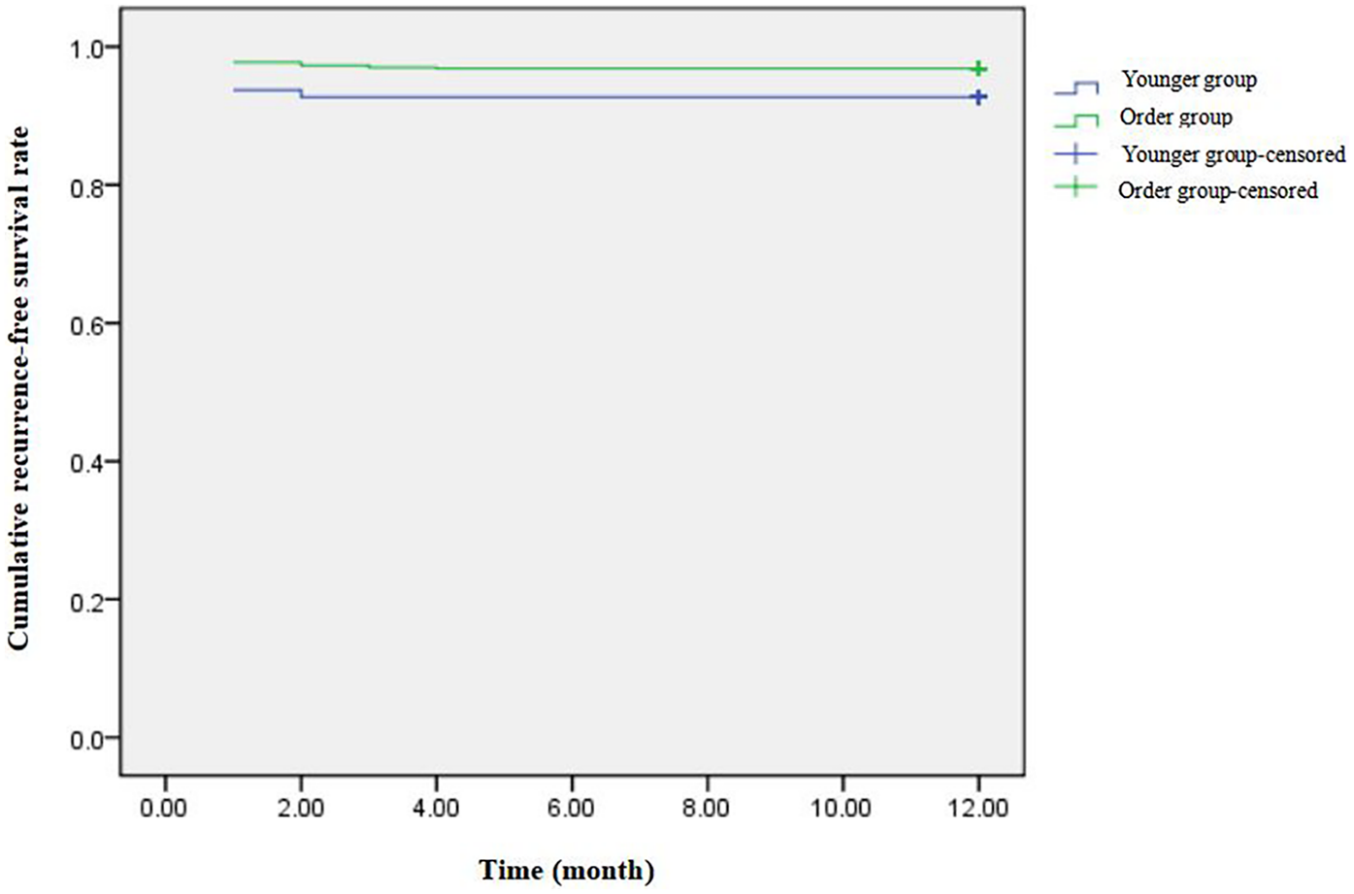
Radiofrequency catheter ablation of ventricular arrhythmias guided by three-dimentional electroanatomic mapping system. This child had PVCs and VT of short duration. An activation sequence mapping method was used to collect the earliest activation points, and the ablation target was the coronal sinus (red dot). During ablation, the temperature control is set at 50°C–55°C, the power is 30–40 W, and the ablation time is 60–120 s.

Procedures were performed under general anaesthesia. Ablation was performed only if PVC/VT was induced. If tachycardia was not induced before ablation, ventricular catheter stimulation and/or isoproterenol induction was used. After femoral venous access, a 4-pole 6 Fr catheter was placed in the right ventricle. A double-curved 4-pole 7 Fr ablation catheter was inserted. Cardiac electrophysiological examination and ablation were performed under x-ray guidance or a three-dimensional mapping system (Ensite NavX, Ensite Velocity, Abbott, Chicago, IL, USA). An activation sequence mapping method was used to collect the earliest activation points. Pacing mapping was used to target patients with rare PVCs. The temperature control mode was used, with targeted power of 30–40 W and a temperature of 50°C–55°C (Figure 1). Acute success was defined as the absence of spontaneous or induced VAs within 45 min.

#### 2.2.3 Follow-up

All patients were followed up at 1, 3, 6, and 12 months after surgery. Electrocardiography, Holter monitoring, and echocardiography findings were reviewed at each follow-up visit. Those lost to follow-up at authors' hospital were followed-up by telephone. Data from hospital visits and telephone calls were also included.

### 2.3 Observation indices

**Acute success rate:** Acute success rate = number of patients with successful RFCA/total number of patients included in the study.

**Recurrence rate:** Recurrence was defined as the occurrence of the same tachycardia observed before RFCA during the follow-up period. Recurrence rate = number of patients with recurrence/total number of patients undergoing RFCA.

**Complications:** Including complications of vascular puncture (local haemorrhage, haematoma, infection, pneumothorax, thrombosis, embolism), catheter operation (aortic regurgitation, myocardial perforation, pericardial tamponade), and discharge ablation (atrioventricular block, myocardial infarction). Patients with suspected complications were examined for early identification.

**Arrhythmia foci:** The ablation target sites were grouped into five categories: the ministry of the septum of the right ventricular outflow tract, free wall of the right ventricular outflow tract, left ventricular outflow tract, left ventricular free wall, coronary sinus, pulmonary valve, right ventricular free wall, tricuspid valve, His bundle region, left anterior branch, left posterior branch, epicardium, right ventricular septum, left ventricular anterior septum, and left ventricular middle/posterior septum.

**Radiation dose:** Radiation dose was defined as the total amount of radiation administered from the beginning to the end of the procedure. The entrance surface dose (ESD) was used as the evaluation standard.

### 2.4 Statistical analysis

Statistical analyses were performed using SPSS version 16.0 (SPSS Inc., Chicago, IL, USA). Normally distributed measurement data are expressed as mean ± standard deviation (SD), whereas non-normally distributed measurement data are expressed as median [interquartile range (IQR)], and count data are expressed as number (percentage). Continuous variables were compared using the unpaired Student's *t*-test or Mann–Whitney *U*-test. Categorical data were compared using the chi-squared or Fisher's exact tests. Differences with *P* < 0.05 were considered to be statistically significant.

## 3 Results

A total of 762 children (mean age, 8.68 ± 3.34 years) with VAs, who underwent RFCA at the authors' hospital were enrolled, including 96 (12.6%) in the younger group and 666 (87.4%) in the older group. All patients were followed-up at the authors' hospital or by telephone.

### 3.1 Basic clinical features

Owing to the different inclusion criteria, the two groups differed significantly in age and body weight (*P* < 0.001). The mean age of the younger group was 3.47 ± 0.75 years, the minimum age was 11 months, and the mean body weight was 16.74 ± 3.98 kg. The mean age of older group was 9.44 ± 2.86 years and the mean body weight was 38.85 ± 15.11 kg. In addition, there were differences in the type of VAs, mapping method, and year of operation between the 2 groups. PVC was the most common type of VA in both groups; however, PVC combined with VT was more common in the younger than in the older group (27.1% vs. 15.3%, respectively; *P* < 0.01). Compared with the older group, the younger age group had an earlier surgical year and more patients underwent x-ray ablation (35.4% vs. 28.6%; *P* < 0.01). There were no significant differences in other basic clinical features between the two groups (Table 1).

**Table 1:** Basic clinical features of children in low age group and older group.

Basic clinical features		Younger group (*n* = 96)	Older group (*n* = 666)	*P*-value
Age (years, mean ± SD)		3.47 ± 0.75	9.44 ± 2.86	*P* < 0.001
Weight (kg, mean ± SD)		16.74 ± 3.98	38.85 ± 15.11	*P* < 0.001
Gender (male/female)		57/39	397/269	*P* = 0.965
Mapping method (x-ray/3D mapping system)		34/62	148/518	*P* = 0.005
Type of VAs (*n*, %)	PVC combined with VT	26 (27.1%)	102 (15.3%)	*P* = 0.009
	PVC	45 (46.9%)	400 (60.1%)	
	VT	25 (26.0%)	164 (24.6%)	
Year of ablation (*n*, %)	2005–2009	32 (33.4%)	134 (20.1%)	*P* = 0.002
	2010–2014	8 (8.3%)	66 (10.0%)	
	2015–2019	48 (50.0%)	324 (48.6%)	
	2020–2022	8 (8.3%)	142 (21.3%)	

PVC: premature ventricular contractions; VAs: Ventricular tachyarrhythmias; VT: ventricular tachycardia.

### 3.2 Arrhythmia foci

The arrhythmic focus is an important factor that affects surgical processes and outcomes. Among the 762 children, four cases involving older children were not induced. Of the 758 remaining cases, 320 originated from the outflow tract (mainly the right ventricular outflow tract), 163 from the septal part, 135 from the inflow tract, while other sites of origin were rare. Fisher's exact test revealed no significant difference in arrhythmia foci between the two groups (*P* > 0.05). Notably, the right ventricular outflow tract was the most common site in younger children (49.9%), followed by the tricuspid annulus (18.8%), whereas the right ventricular outflow tract was the most common site in older children (40.9%), followed by the left ventricular septum (21.1%) (Table 2).

**Table 2:** Arrhythmia foci of children in younger group and older group.

Arrhythmia foci		Younger group (*n* = 96)	Older group (*n* = 666)	*P*-value
Outflow tract (*n*, %)	Septum of right ventricular outflow tract	39 (40.6%)	211 (31.7%)	*P* = 0.121
	Free wall of right ventricular outflow tract	9 (9.3%)	61 (9.2%)	
	Left ventricular outflow tract	0	14 (2.1%)	
	left ventricular free wall	1 (1.0%)	19 (2.9%)	
	Coronary sinus	1 (1.0%)	37 (5.6%)	
	Pulmonary valve	2 (2.1%)	3 (0.5%)	
	Right ventricular free wall	8 (8.3%)	29 (4.4%)	
Inflow tract (*n*, %)	Tricuspid valve	18 (18.8%)	94 (14.1%)	
	HIS bundle region	2 (2.1%)	21 (3.2%)	
Branch (*n*, %)	Left anterior branch	0	10 (1.5%)	
	Left posterior branch	1 (1.1%)	12 (1.8%)	
Epicardium (*n*, %)	Epicardium	0	3 (0.5%)	
Septum (*n*, %)	Right ventricular septum	0	8 (1.2%)	
	Left ventricular anterior septum	1 (1.1%)	15 (2.3%)	
	Left ventricular middle/posterior septum	14 (14.6%)	125 (18.8%)	
Not induced (*n*, %)	Not induced	0	4 (0.6%)	

注, 计数资料表示为数量 (百分比).

### 3.3 Acute success rate, complications, and recurrence rate

**Figure 2:**
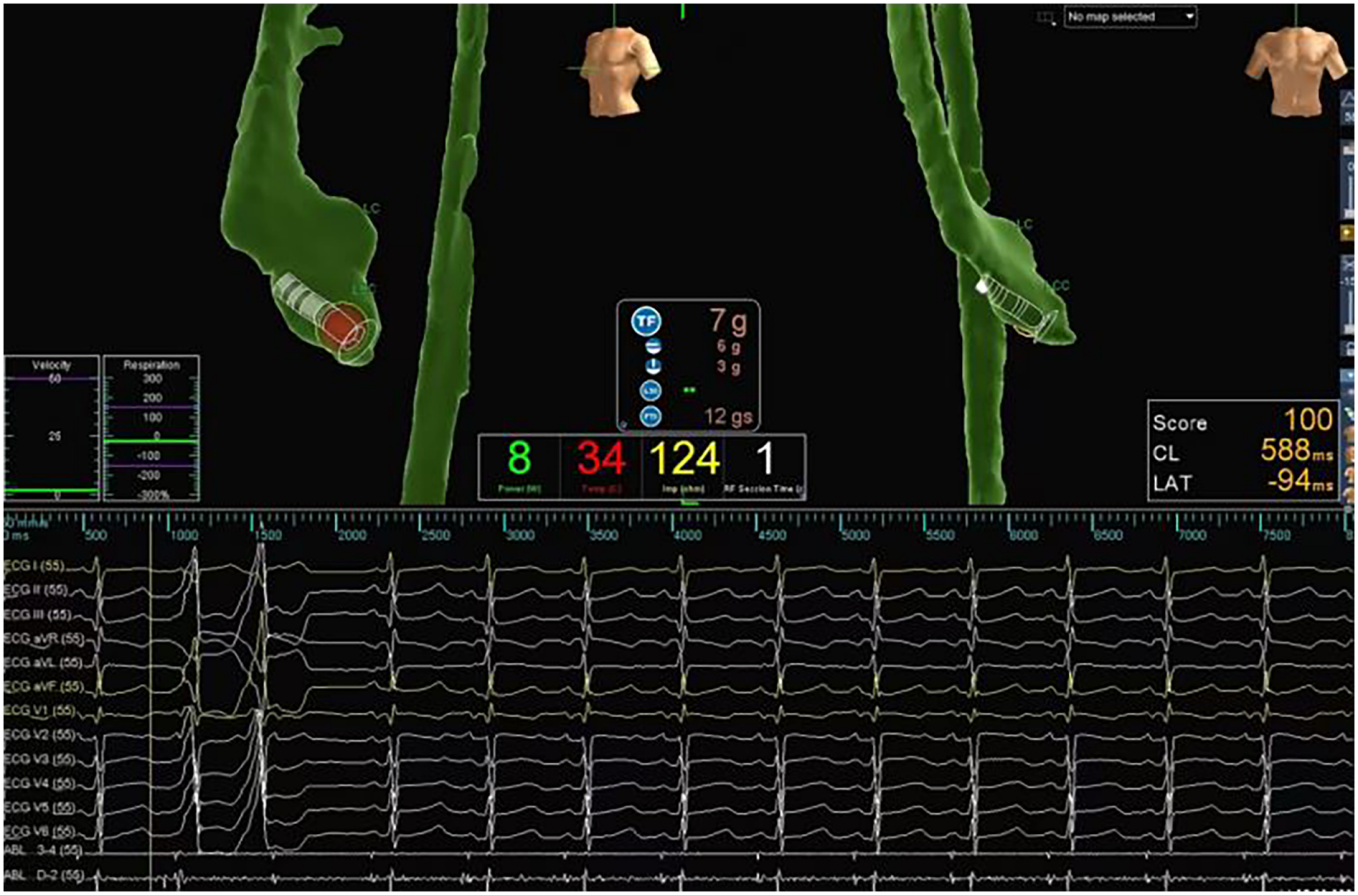
The recurrence-free survival curves for two groups.

Acute success was achieved in 95 of 96 cases (99%) in the younger group, and only one child terminated the operation due to severe heart failure. The acute success rate in the older group was 98.6%. VAs were not successfully induced in four cases, and the operation was terminated in five cases due to poor cardiac function and circulatory instability. Two children succeeded in secondary ablation, and the rest of them continued taking antiarrhythmic drugs. In the younger group, complications occurred in two (2.1%) cases, including one case of left anterior branch block and one case of incomplete right bundle branch block. Incomplete right bundle branch block occurred in two (0.3%) patients in the older group. All complications were persistent and self-cured during follow-up. No deaths occurred, and there were no significant differences in the acute success and complication rates between the two groups. VAs recurrence after acute successful ablation occurred in 28 children. Ten children underwent secondary ablation, of which 9 children were free of VAs at the end of the follow-up. A 3-year-old girl relapsed again, and her VAs was cured during the third ablation. The remaining 18 children received only drug treatment. The recurrence rate was higher in the younger group vs. the older group (7.3% vs. 3.2%, respectively; *P* < 0.05) (Table 3 and Figure 2). Considering that differences in mapping methods may affect the recurrence rate, patients were stratified according to mapping method and analysis revealed no statistical difference in the recurrence rate between the two groups (*P* > 0.05).

**Table 3:** Observation indexes after RFCA in low age group and older group.

Observation indexes	Younger group (*n* = 96)	Older group (*n* = 666)	*P*-value
Acute success (*n*, %)	95 (99.0%)	657 (98.6%)	0.999
Complications (*n*, %)	2 (2.1%)	2 (0.3%)	0.081
Recurrence (*n*, %)	7 (7.3%)	21 (3.2%)	0.044
ESD [mGy, Median (IQR)]	0.1 (47.5)	0 (12)	0.036

ESD, entrance surface dose; RFCA, radiofrequency catheter ablation.

### 3.4 Radiation dose

The ESD between the two groups exhibited significant differences (*p* < 0.05), 0.1 (47.5) mGy and 0 (12) mGy, respectively. Due to the significantly higher x-ray exposure associated with conventional fluoroscopic ablation compared with three-dimensional mapping guided ablation (*P* < 0.001), patients were stratified according to mapping method, which revealed no statistical difference in radiation dose between the two groups (*P* > 0.05) (Table 3).

## 4 Discussion

The key findings of this retrospective study were as follows. First, PVC was the main arrythmia type in both the younger and older groups; however, the younger group had more PVC combined with VT. Second, the younger group had earlier ablation years and more patients underwent x-ray ablation. Third, the right ventricular outflow tract was the most common arrhythmia focus in both groups, followed by the tricuspid annulus in the younger group and the left ventricular septum in the older group. Fourth, the acute complication rates were similar between the two groups. The recurrence rate and radiation dose were higher in the younger age group than in the older group; however, there was no significant differences after stratification according to mapping method.

VAs included PVC and VT. PVC has the possibility of spontaneous regression and is often regarded as a benign arrhythmia in children with normal heart structure (3, 4). Uysal et al. (5) followed 226 children with PVC for 8.7 ± 3.2 years, and found that most had a good prognosis. There was no significant difference in the PVC improvement rate between the treated and untreated groups, suggesting that medical treatment may not be necessary in children with PVC. However, frequent PVC may lead to tachycardia-induced cardiomyopathy and abnormal cardiac function. Spector et al. (6) reported tachycardia-induced cardiomyopathy in 19.4% of children with frequent PVC. However, in another study by Abadir et al. (7), 15% of children with frequent PVC exhibited mild left ventricular systolic dysfunction associated with a shorter interictal interval. The children in our study had PVC combined with VT; however, recent studies have suggested that a high PVC burden (>20%) is not related to VT (4). VT is mostly idiopathic and has a good prognosis in children without structural heart disease(s). Simultaneously, the possibility of spontaneous VT regression in younger children was significantly higher than that in older children. Studies have shown that approximately 89% of infants and young children experience spontaneous regression of VT compared with only 56% of older children (8). Sustained VT can lead to cardiac arrest and potentially life-threatening arrhythmias. In a long-term study of 129 children with VT by Şengul et al. (9), three children (including one infant) experienced sudden death, 11 experienced life-threatening events (such as syncope and cardiac arrest), and five developed tachycardia cardiomyopathy. Therefore, even in children with a normal heart, frequent PVC and VT should be considered and timely intervention should be provided. RFCA is an interventional technique. All children enrolled in this study fulfilled the relevant criteria and underwent RFCA. In our study, the most common arrhythmia focus in the VAs was the right ventricular outflow tract, which is consistent with a previous study (3). However, in younger children, the VAs originated more often from the tricuspid valve than from left ventricular septum.

The safety and effectiveness of RFCA in the treatment of VAs in children have been confirmed (10). However, younger children have smaller heart volumes and blood vessel diameters, which make RFCA more difficult. According to the 2019 Japan Circulation Society/Japanese Heart Rhythm Society guidelines regarding the nonpharmacological treatment of arrhythmias (1), RFCA is only recommended for infants with life-threatening arrhythmias and drug resistance, and this procedure should be performed by experienced paediatric electrophysiologists. Among the 762 children who underwent RFCA in the present study, there were more PVCs combined with VTs in the younger age group than in the older age group, which may be related to the different indications for RFCA. The severity of VT was more pronounced in the younger age groups, with many cases being persistent and resistant to drug treatment.

In terms of surgical outcomes, the acute success and complication rates for RFCA in the younger age group were 99.0% and 2.1%, respectively, which were comparable to 98.6% and 0.3%, respectively, in the older group. Complications were mild in both groups, and no surgery-related deaths occurred. The recurrence rate was higher in the younger vs. older age group (7.3% vs. 3.2%); it may be related to incomplete lesion formation, procedural difficulty, or anatomical differences. And the difference was not statistically significant after excluding confounding factors. Results of this study suggest that RFCA is safe and efficacious for the treatment of VAs in young children, which are partially consistent with the results of previous studies. Another domestic study of 328 children with VAs who underwent RFCA included 20 infants ≤ 3 years of age, with an acute success rate of 94.7%, a recurrence rate of 22.2%, and one case of intraoperative vascular complications. That study did not compare infants with older children, and the overall acute success rate was 88.3%, with a recurrence rate of 15.5%, and complication rate of 1.2% (11). The recurrence and complication rates of infants were higher than those of older children but within the acceptable range. In a study involving 53 children with VAs, Wu et al. (12) reported that the acute success rate of RFCA was 97%, with a recurrence rate of 8.5%, and two patients developed postoperative thromboembolism. Three infants were included, all of whom underwent successful ablation without complications; however, two experienced recurrence, suggesting that the recurrence rate may be higher in younger children. In addition, some case reports have described RFCA in young children with Vas (13, 14). No serious complications occurred and patient quality of life improved.

In Yuan Lin's adult-oriented study, atrioventricular block was the most common complication after RFCA for VT (15). Although the complications in this study were only transient branch blocks, in other children-oriented studies, vascular puncture-related complications were more common, possibly due to their smaller blood vessels. Besides oral anticoagulation before ablation, ultrasound-guided vascular puncture could significantly reduce such complications (16), and may be applicable for vascular puncture in younger children. Additionally, pericardial effusion is a serious complication after radiofrequency ablation. Given that children have smaller hearts, precise positioning during the procedure, controlled ablation energy, and continuous monitoring of vital signs are essential to prevent such complications. Previous reports have documented fatalities resulting from complications following RFCA of VAs. One case involved a child with VT who experienced fibrillation and cardiac arrest in recovery room. An emergent angiography was done which confirmed total occlusion in the left main coronary artery. Coronary balloon angioplasty was performed emergently but failed to rehabilitate the child, who expired after 1 day (10). In young children, ventricular tachycardia may resolve spontaneously and RFCA has certain risks. 2015 European Society of Cardiology (ESC) Guidelines recommended that asymptomatic children with frequent isolated PVCs or an accelerated ventricular rhythm and normal ventricular function be followed-up without treatment. Medical therapy or RFCA is recommended in children with frequent PVCs or VT thought to be causative of ventricular dysfunction. Consequently, it is essential to conduct a thorough evaluation of the child's condition, the efficacy of pharmacological therapy, and the complexity of radiofrequency ablation prior to determining whether RFCA or medical therapy or wait-and-see approach is more appropriate (17).

Children (especially infants) are still in the growth stage and are more sensitive to x-ray radiation, which may lead to malignant tumours (18). Therefore, x-ray exposure should be reduced as much as possible during RFCA. Currently, three-dimensional mapping systems are widely used in RFCA, and a large number of studies have confirmed their safety and effectiveness (19–21). Since 2011, our hospital has gradually transitioned from traditional x-ray fluoroscopy to a three-dimensional mapping system. To maintain a high success rate and low incidence of complications, x-ray exposure has been significantly reduced. Compared with the older group, more children in the younger age group underwent x-ray ablation and received higher radiation doses. One reason for this may be that the 2002 expert consensus on paediatric radiofrequency ablation did not delineate a clear weight boundary (22). Another reason may be the poor efficacy of anti-arrhythmic drugs in the early stages, and RFCA is performed in younger children due to ineffective drug therapy. There were no significant differences between the two groups after stratification according to mapping method.

Our study had some limitations, the first of which was its retrospective design. Second, there was a selection bias in only including surgical patients. As a result, conclusions regarding VA foci in this study can only reflect the situation in the surgical patient population but cannot completely represent the actual population. Furthermore, our study was conducted over a longer time span than previous studies. During the study period, our hospital gradually transitioned from x-ray fluoroscopy to three-dimensional mapping; however, in the interim, operator technologies have also improved, which may have caused a corresponding bias.

## 5 Conclusions

RFCA was safe and effective for the treatment of VAs in younger children but was not significantly different from that in older children. However, considering the possibility of spontaneous VA regression in younger children, the indications for RFCA should be strictly controlled. RFCA is recommended only in young children with drug-resistant VAs and should be performed by experienced electrophysiologists. In addition, reducing x-ray exposure is highly recommended.

## Data Availability

The raw data supporting the conclusions of this article will be made available by the authors, without undue reservation.
